# Dysregulated MicroRNAs in Chronic Lymphocytic Leukemia

**DOI:** 10.7759/cureus.68770

**Published:** 2024-09-06

**Authors:** Oana Mesaros, Stefana Veres, Madalina Onciul, Emilia Matei, Laura Jimbu, Alexandra Neaga, Mihnea Zdrenghea

**Affiliations:** 1 Hematology, Iuliu Hatieganu University of Medicine and Pharmacy, Cluj-Napoca, ROU; 2 Hematology, Ion Chiricuta Oncology Institute, Cluj-Napoca, ROU; 3 Otolaryngology, Policlinica Grigorescu, Cluj-Napoca, ROU; 4 Pathology, Ion Chiricuta Oncology Institute, Cluj-Napoca, ROU; 5 Hematology, Oncology Institute "Prof. Dr. Ion Chiricuta", Cluj-Napoca, ROU

**Keywords:** chronic lymphocytic leukemia, dysregulated mir, micrornas, predictive markers, prognostic markers

## Abstract

MiRNAs are a class of non-coding RNAs acting as gene expression regulators by modulating the lifespan of messenger RNA. Commonly referred to as the most frequent leukemia in the Western world, chronic lymphocytic leukemia (CLL) is a lymphoproliferative malignancy characterized by clonal expansion of CD19, CD23, and CD5-positive mature B-cells. While this pathology is regarded as less aggressive and has a variety of treatment options, the cause of its clinical heterogeneity is not yet understood. Moreover, the prognostic markers and treatment recommendations based on predictive markers are limited. This review aims to investigate some miRNAs that are dysregulated and possibly involved in CLL pathogenesis as a starting point for the proposal of new prognostic and predictive markers and, as more agents targeting miRNA expression become available, their potential role as therapeutic targets.

## Introduction and background

Chronic lymphocytic leukemia (CLL)/small lymphocytic lymphoma (SLL) is a biologically and clinically heterogeneous hematologic malignancy of the elderly that is slightly more frequent in male patients, with a median age at diagnosis around 72 years old [1,2]. Nonetheless, with the rising prevalence of routine blood tests, it appears that an increasing number of young individuals, even those under 55 years of age, are being diagnosed with CLL at early, asymptomatic stages [3]. Even though CLL’s clinical evolution and therapeutical approach are extremely different from those of acute leukemias, from an epidemiological point of view, they are conventionally included together, which makes CLL the most common leukemia in the Western world, being responsible for over 30% of all cases of leukemias [4]. Due to its frequent indolent course, some authors consider that the incidence of CLL should be calculated independently and not included with the rest of the acute leukemias.

CLL is characterized by the clonal expansion of mature B-cells that are CD5+, CD19+, and CD23+. These cells proliferate and accumulate in the bone marrow, lymphoid tissues, and peripheral blood [2]. The diagnostic accuracy is crucial, particularly because other lymphoproliferative diseases may have similar immunophenotypes but require different treatment strategies. In 1994, Estella Matues proposed a scoring system for the diagnosis of CLL, “Matutes Scores" (MS), which initially included the evaluation of surface membrane immunoglobulin (SmIg) weak, CD5, CD19, CD23, CD22, and FMC7 [5]. CD22 was later replaced by CD79b [6]. Depending on the presence or absence of the evaluated markers, each is assigned a score between 1 and 0. A minimum score of 4 is required for a diagnosis of certainty. Exceptions are atypical CLL cases for which additional markers will be added [7]. Other markers, such as CD 200, have been proposed to be included in the MS, but for the time being it remains unchanged.

The pathogenesis of CLL is not completely understood; however, it is associated with impaired immune responses and abnormalities in genetic and cellular signaling pathways. These factors ultimately contribute to immune evasion, resistance to apoptosis, and uncontrolled proliferation of malignant cells. Among other factors, microRNAs (miRNAs) influence the expression level of the genes involved in CLL development, progression, and resistance to treatment, acting like oncogenes or tumor suppressor genes [8].

This review aims to evaluate the most important miRNAs dysregulated in CLL, compile this information, and identify new predictive and prognostic markers, which will lead to the discovery of new therapeutic targets.

## Review

MiRNAs

MiRNAs are short, non-coding RNA molecules, typically consisting of approximately 22 nucleotides, that play a role in gene expression modulation by enhancing or inhibiting it [9]. In 1993, Lee’s team isolated lin-4, the first miRNA ever described, using the Caenorhabditis elegans organism as a model. By proving that lin-4 could downregulate lin-14 messenger RNA (mRNA) and lower protein synthesis, they brought the field of molecular biology into a new age [10]. Seven years later, in 2000, Reinhart et al. discovered the second miRNA in nematodes and the first human miRNA, lethal-7 (let-7), that would regulate lin-41 mRNA [11,12]. Further studies proved that in humans, let-7 miRNA is a family comprised of 10 different non-coding molecules [13].

Complementary research indicated that miRNAs hold an essential role in the physiological development of all beings, including the human body, regulating key processes like embryogenesis, cellular cycle, cell differentiation, proliferation, and apoptosis [14-16]. Nonetheless, dysregulations of miRNAs could lead to the development of certain diseases, including malign hemopathies, as Calin et al. concluded in 2002 when they discovered that miR-15 and miR-16 are downregulated in more than 65% of CLLs [17].

MiRNAs are synthesized via either the canonical or non-canonical pathway, which involves the presence of RNase III endonucleases Drosha and/or Dicer, along with certain members of the Argonaute (AGO) protein family.

**Figure 1:**
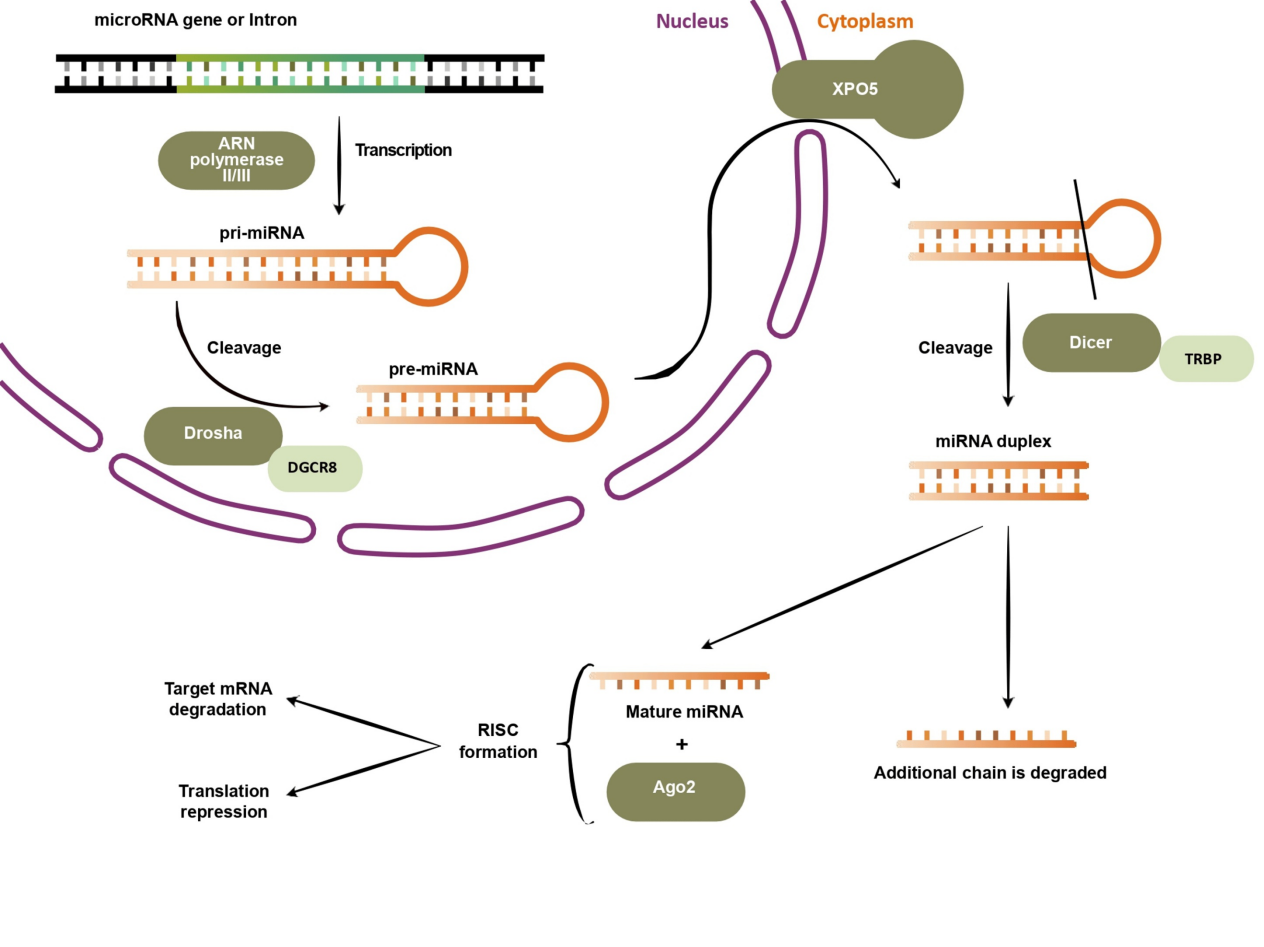
The canonical pathway of miRNAs biogenesis Inside the nucleus, pri-miRNA is formed as the miRNA genes or Introns are transcribed by RNA polymerase II or III. The pri-miRNA is cleaved by the Microprocessor Complex (Drosha and DGCR8 proteins), forming the pre-miRNA, which is exported by Exportin 5 into the cytoplasm, where the Dicer protein, in association with TRBP, cleaves it, resulting in a miRNA duplex. One of the strands will be loaded into AGO2 and form RISC, which will be involved in mRNA regulation. The spare strand will be degraded [9,19,20]. miRNAs: MicroRNAs; pri-miRNAs: primary miRNAs; RISC: RNA-induced silencing complex; TRBP: transactivation response RNA binding protein Image credit: figure created by authors

Most miRNAs are generated through the canonical pathway (Figure 1). They start as intron-transcribed primary miRNAs (pri-miRNAs), which are then converted into precursor miRNAs (pre-miRNAs), transported to the cytoplasm, and processed into short duplexes. These miRNA duplexes interact with AGO family members and other proteins, leading to the formation of the RNA-induced silencing complex (RISC) that targets a specific mRNA, causing translation inhibition and gene silencing [9]. The non-canonical pathway, on the other hand, is less studied and does not always follow the same sequence of steps, as it bypasses at least one of the previously described stages [9,18].

MiRNAs upregulated in CLL

MiR-223

MiR-223 is located on the X chromosome, and it was first described as a regulatory protein for stem cell differentiation and immune responses [21,22]. In neoplastic processes, miR-223 contributes to the modulation of various signaling pathways, including the NF-κB and PI3K/AKT pathways. By targeting different genes involved in these pathways, miR-223 influences the cellular response to stress and apoptosis, affecting the overall leukemic burden [23-25]. Additionally, the role of miR-223 in the tumor microenvironment merits attention. It has been suggested that CLL cells regulate miR-223 expression in the neighboring stromal cells, potentially affecting the cellular interactions that support tumor growth and survival [26].

In CLL, downregulation of miR-223 is associated with poor prognosis, suggesting it could be used as a predictive marker [27]. Starting from the fact that heat shock proteins (HSPs) are upregulated in cancer, including in CLL, Rodríguez-Vicente et al. found that there are cases of CLL that have a polymorphism that prevents miR-223 from binding to HSP90B1, which will lead to overexpression of B lymphocytes and more aggressive disease. Modulation of miR-223 could be used as a therapeutic target [28].

MiR-223’s expression is increased in healthy controls compared to CLL patients [29]. Its expression was inversely correlated to progression from Binet A (RAI 0) to C (RAI III-IV) and with STAT3 expression [29]. Its downregulation was observed in patients with unmutated immunoglobulin heavy chain variation (IgHV) and high expression of ZAP70. MiR-223’s overexpression was correlated with the presence of del13q, making this miRNA a possible prognostic marker in CLL [30-32].

MiR-29

This family includes miR-29a/miR-29b-1, encoded on 7q32.3 chromosome, and miR-29b-2/ miR-29c located on 1q32.2 chromosome [33]. MiR-29 family members, particularly miR-29a, miR-29b, and miR-29c, are downregulated in CLL, potentially contributing to the pathogenesis and progression of the disease [34-36]. The downregulation of miR-29 in CLL is associated with the upregulation of its target genes, several of which are involved in pathways related to cell survival, proliferation, and apoptosis resistance. This imbalance results in enhanced survival of neoplastic cells, thereby facilitating the accumulation of malignant lymphocytes in the blood and bone marrow [37-41].

MiR-29 overexpression is associated with an indolent course of CLL, and additionally, lower levels of miR-29 have been correlated with poorer prognosis in CLL patients, reflecting the miRNA's role as a potential tumor suppressor [36]. MiR-29 up-regulates the oncogenic T-cell leukemia/lymphoma 1 (TCL1) gene, which triggers numerous signaling pathways, including NF-kB and Pi3K. Overexpression of TCL1 stimulates lymphomagenesis and tumor progression in many lymphoproliferative diseases, including CLL [42]. The MiR-29 expression was inversely correlated with TCL1 expression [37]. The interaction between miR-29 and other signaling molecules in CLL is also a subject of intense scrutiny, which could help elucidate the complex networks that drive malignancy. Additionally, strategically targeting miR-29 pathways could create new opportunities for clinical management, offering patients tailored treatment options. Overall, the connection between CLL and miR-29 presents a promising path for enhancing our understanding and treatment of this common blood cancer.

MiR-34

This family includes miR-34a, located on 1p36. 22 chromosome, miR-34b and miR-34c located on 11q23. 1 chromosome [43]. MiR-34 is known for its role as a tumor suppressor and is frequently downregulated in various cancers, including CLL [44].

The expression of miR-34a is often reduced in CLL patients, correlating with a poor prognosis, shorter treatment-free survival, and can predict an evolution towards Richter’s Syndrome [32,45]. This downregulation can result from the presence of TP53 deletion, which is a crucial regulator of miR-34a expression [43,46,47]. TP53 mutations are common in CLL and contribute to resistance to chemotherapy and disease progression. MiR-34 demonstrates regulatory effects on other pathways, including the silent information regulator 1 (SIRT1), CDK4, CDK6, CCND1, and CCNE2, and can modulate cellular growth and survival [48,49].

MiR-34 might have a role as a predictive marker, as its downregulation is associated with resistance to Fludarabine, by inhibiting the transcription factor Forkhead box P1 (FOXP1) and lowering its potential to upregulate BCR signaling [50].

MiR-155

This was initially identified as the B-cell integration cluster (BIC) gene and is encoded on the 21q21.3 chromosome [51]. MiR-155 is essential for B-cell maturation and function, modulating the germinal center response and formation of class-switched plasma cells, but it also plays an important part in hematopoiesis [52-54]. In addition, it exerts a pro-tumorigenic effect in hematological malignancies, such as CLL [55].

Compared to healthy subjects, miR-155 has been observed to be upregulated in patients with monoclonal B-cell lymphocytosis (MBL) and even more in CLL ones [56,57]. Moreover, miR-155 expression assessed in the plasma of pre-treatment CLL patients was lower in those who achieved a good response to treatment compared to non-responders, which indicates that miR-155 could be a valuable predictive and prognostic marker in CLL [56].

The overexpression of miR-155 is associated with ZAP-70 expression, unmuted IGHV, and 17q or 11q deletions and thus correlated with more aggressive disease progression [55,56,58,59].

MiR-181

This family includes miR-181a1 and miR-181b1, which are localized on the first chromosome miR-181a2 and miR-181b2, localized on the ninth chromosome; and miR-181c and miR-181d which are located on the 19th chromosome [60,61]. MiR-181 has been implicated in apoptosis, cell cycle regulation, and tumor microenvironment modulation [62]. Along with miR-29, miR-181 expression is inversely correlated with TCL1 oncogene expression [37].

Moreover, miR-181, which acts as a regulator for B-cell differentiation, is decreased in CLL, leading to an upregulation of the target proteins MCL1 and BCL2, which will induce resistance to treatment [63]. It has also been observed that miR-181b expression decreases with disease progression, while the ones of MCL1 and BCL2 increase [64,65].

Downregulation of miR-181 is associated with disease progression and resistance to apoptosis, suggesting its role as a prognostic marker [65]. In 2011, Visone et al. discovered that miR-181b could be a biomarker for disease progression in CLL. They examined samples from 114 patients with either progressive or stable disease. Results revealed that miR-181b is the most significantly dysregulated miRNA in the group with progressive disease compared to the stable disease group [64]. In 2021, Di Marco et al. exploited miR-181b as a therapeutic target in CLL and concluded that increasing the expression of miR-181b in CLL patients leads to CLL cell death in vitro, suggesting a novel and challenging treatment approach for CLL patients [65].

MiR-17/92 Cluster

MiR-17/92 cluster was discovered in 2004 and is encoded on 13q31. 3 chromosome and is a cluster comprising of miR-17, miR-18a, miR-19a, miR-19b-1, miR-20a, and miR-92a-1, also known as oncomiR-1 [66-69]. This family of miRNAs plays a pivotal role in the regulation of various biological processes, including cell growth, survival, and differentiation [60,70].

It is frequently dysregulated in solid and hematological cancers and has been shown to promote cell proliferation by targeting pro-apoptotic factors such as Bim and Fas [71,72]. B-cell receptor (BCR) upregulates this family of miRNAs in aggressive CLLs with unmutated IGHV, which in return will downregulate antiproliferative and proapoptotic genes, suggesting that this cluster of miRNAs could be used as a prognostic marker [73,74].

Evaluated in different CLL risk groups, the expression of the members of this cluster was found to be heterogeneous. MiR-18a-5p, miR-19b-1-5p, and miR-92a-1-5p expressions did not differ among patients, but that of miR-17-5p was directly proportional with disease stage and associated with a worse prognosis, whereas miR-19a-3p and miR-20a-5p were inversely correlated [73,75,76].

MiR-145

MiR-145 is located on the fifth chromosome and is part of the miR-143/miR-145 cluster [75]. It was discovered to be overexpressed in the serum of patients with CLL in comparison to the serum of healthy individuals. It acts on PTEN, NOTCH1, NOTCH1, CDKN1, and MYC, impacting cell division and apoptotic processes [75].

A study performed on the sera of 80 CLL patients showed that the miR-145-5p strand of the miR-145 duplex acts as a tumor suppressor. Its upregulation results in a reduction of APRIL expression, a molecule that is seldom found in tissues other than tumor tissue. APRIL acts as an oncogene, influencing the NF-kB signaling pathway. Upregulation of miR-145-5p leads to downregulation of APRIL, decreasing tumor proliferation rate, and stimulating apoptotic processes [77]. Considering the tumor-suppressive function of miR-145 in CLL, approaches designed to restore its expression could hold therapeutic effects.

MiR-650

MiR-650 is located on the 22nd chromosome [78]. The over-expression of miR-650 has been associated with a favorable CLL prognosis. It can be considered a predictive survival and time-to-treatment marker [79]. It is presumed that miR-650 could influence CLL through multiple pathways, including the modulation of apoptosis, cell cycle regulation, and immune evasion. However, some conflicting study results are found in the literature, where the miR-650 over-expression correlates with advanced disease stages (Binet B/C), p53 gene aberrations, and unmutated IgHV status. MiR-650 has been inversely correlated with NMYC downstream-regulated gene-2 (NDRG2) expression, a well-known tumor suppressor, associating miR-650 expression with a worse outcome in CLL [80]. MiR-650 is emerging as an important player in the pathogenesis of CLL. Its dysregulation contributes to the malignant behavior of CLL cells, while its prospective role as a therapeutic target warrants further investigation.

A summary of the miRNAs upregulated in CLL is provided in Table 1.

**Table 1:** miRNAs upregulated in CLL miRNAs: MicroRNAs; CLL: chronic lymphocytic leukemia

miRNA	Location on chromosomes	Expression in CLL	Possible prognosis
miR-223 [21,27]	X	Upregulated in early stages CLL	Good while upregulated
miR-29 [33,36]	1 and 7	Upregulated in indolent CLL	Good while upregulated
miR-34 [32,43,45]	1 and 11	Upregulated	Good while upregulated
miR-155 [51,55]	21	Upregulated	Bad while upregulated
miR-181 [37,61]	1, 9, and 19	Upregulated	Bad while upregulated
miR-17/92 [66,73]	13	Upregulated	Bad while upregulated
miR-145 [75,77]	5	Upregulated	Good while upregulated
miR-650 [78,80]	22	Upregulated	Good while upregulated

MiRNAs downregulated in CLL

MiR-15a/MiR-16-1

As already mentioned, miR-15a/miR-16-1 represents the “archetypes” of non-coding ARNs involved in cancer pathogenesis. They are located on the 13q14 chromosome and are downregulated in 2/3 of CLLs. The 13q14 deletion is present in 50-60% of all CLL cases and its presence has been correlated with the downregulation of miR-15a/miR-16-1. Another interesting aspect is the length of the mutation, as it has been shown that a larger deletion would cause a more severe downregulation. Nonetheless, miR-15a/miR-16-1 downregulation has been observed even without 13q14 deletion. Further investigation suggested that their downregulation in CLL could second a microdeletion in the miR-15-a/miR-16-1 locus or even the presence of a germline mutation in their primary transcript [17,81-84]. The miR-15a/miR-16-1 cluster presents promising avenues for clinical application, particularly as a biomarker for CLL. The deletion or downregulation of this cluster in CLL has been shown to correlate with more aggressive disease and poorer prognosis, indicating potential value in patient stratification [85].

MiR-15a/miR-16-1 target multiple oncogenes and even tumor suppressor genes, with roles in tumorigenesis, apoptosis, regulation of growth and cell cycle, and one of the most important is BCL-2, known for its anti-apoptotic function [85,86]. The loss of MiR-15a/miR-16-1 was correlated not only with high BCL-2 but also with higher levels of TP53 [87]. Applying the information in vivo, in 2012 Kasar et al. showed that systemic delivery of miR-15a/miR-16-1 stimulates a reduction in malignant B-1 cells in the mouse models [88]. Another therapeutic approach was evaluated in malignant pleural mesothelioma, where the restoration of miR-15a/miR-16-1 expression in xenograft-bearing nude mice inhibited tumor growth [89].

MiR-708

MiR-708 is located on the 11th chromosome and is downregulated in CLL, compared to healthy subjects [90,91]. There aren’t many studies about miR-708 in CLL, but it has been suggested that it could function as a tumor suppressor by inhibiting CD38 and NOTCH1, which are adverse prognostic markers [91]. Furthermore, it modulates the NF-kB pathway by suppressing the inhibitor of nuclear factor kappa-B kinase subunit beta (IKKβ), which is overexpressed in CLL [90].

An under-expression of miR-708 has been correlated with a worse prognosis and a shorter time to treatment [91].

Upregulation of miR-708 was inversely correlated with activation of the NF-kB pathway, making it a formidable therapeutic target in CLL [90].

MiR-138

MiR-138 is a family consisting of miR-138-1 localized on chromosome 3p21.3, and miR-138-2, localized on chromosome 16 [92]. MiR-138 directly targets and suppresses the expression of the enhancer of zeste homolog 2 (EZH2) gene in non-small cell lung cancer. EZH2 is known to function as a transcriptional repressor that promotes cell proliferation and survival via the methylation of histones, and in CLL was associated with a more aggressive disease [93]. In CLL, EZH2 inhibition has an anti-tumor effect, which suggests that miR-138 is a potential therapeutic target [94]. Besides that, miR-138 influences CD-95-mediated apoptosis and stimulates this process by increasing BAX and decreasing BCL-2, which will activate caspase-3 and lead to cell death [92]. MiR-138 downregulates APT1 protein expression, which was found to be overexpressed in CLL and correlated with decreased CD-95-mediated apoptosis [95]. MiR-138 is downregulated in CLL [96].

MiR-9-3

MiR-9-3 is part of the miR-9 family, along with miR-9-1 and miR-9-2, and is located on the 15th chromosome [97]. It acts on the NF-kB signaling pathway, holding a tumor suppressor effect. An increased level of miR-9-3 will lead to decreased tumor proliferation and increased apoptosis [97].

Hadi et al. studied the expression miR-9 in CLL, which was downregulated, in correlation with transforming growth factor beta receptors 1 and 2 (TGFBR1 and TGFBR1). TGFB plays a critical role in physiological hematopoiesis. By targeting TGFBR1, miR-9 modulates the TGFB axis and could reinduce normal hematopoiesis, suggesting a possible target for new therapeutical approaches. In CLL miR-9 was inversely correlated with TGFBR1 [98].

MiR-143

MiR-143 is located on the fifth chromosome [99]. Its expression was evaluated in leukemia cells, in patients diagnosed with CLL, acute myeloid leukemia (AML), acute lymphoblastic leukemia (ALL), and chronic myelocytic leukemia (CML) and, in comparison to healthy controls, was downregulated and inversely correlated with DNA methyltransferase 3A (DNMT3A) [100]. Downregulation of DNMT3A results in a decreased tumor proliferation rate and apoptosis, indicating that miR-143 could be a potential therapeutic target [100]. Furthermore, miRNA-143 targets the extracellular signal-regulated kinase 5 (ERK5), which plays a role in cell proliferation and differentiation. Silencing of this miRNA can dysregulate ERK5 signaling and increase tumor cell proliferation. MiR-143 can also inhibit tumor growth by downregulating c-myc oncogene expression via p53 [101].

MiR-143 was found to be under-expressed in a proportion of CLL patients with 13q deletion, which is a known positive prognostic factor. However, this has only been observed in patients with a high burden of del13q, which seems to confer a reserved prognosis: a higher number of CLL cells possessing del13q was correlated with a lower miR-143 expression and a more adverse prognostic [102-104].

MiR-143 downregulation in CLL cells supports its classification as a tumor suppressor, and its targeting of key signaling pathways highlights its potential as a therapeutic target.

MiR-126

MiR-126 is located on the ninth chromosome. In general, miR-126 targets PI3K and has a tumor-suppressor effect, modulating angiogenesis, tumor growth, and metastasis [105,106]. It is decreased in CLL cells compared to B cells of healthy subjects. A decrease is correlated with disease progression [63].

MiR-126 expression was evaluated in patients with CLL before and during ibrutinib treatment, in relationship with p85β, a regulatory subunit of the phosphatidylinositol- 4,5- bisphosphate 3- kinase (PI3K) and with epidermal growth factor-like domain 7 (EGFL7), which is the host gene of this miRNA [107]. Firstly, Guinn et al. found that the expression of miR-126 was negatively correlated with p85β in both groups of patients and that after ibrutinib treatment, miR-126 was positively correlated with EFFL7, suggesting that both, miR-126 and the host gene might have similar promoters. It was also observed that after one month of treatment with ibrutinib, miR-126 expression increased [108].

MiR-126 plays a critical role in modulating apoptosis and cell proliferation in CLL. The reduction of its expression in CLL cells reinforces its classification as a tumor suppressor, which has important implications for understanding the biology and progression of the disease.

Table 2 offers a summary of the miRNAs that are downregulated in CLL.

**Table 2:** miRNAs downregulated in CLL miRNAs: MicroRNAs; CLL: chronic lymphocytic leukemia

miRNA	Location on chromosomes	Expression in CLL	Possible prognosis
miR-15a/miR-16-1 [17,81-84]	13	Downregulated in 2/3 of cases	Good while downregulated
miR-138 [92,96]	3 and 16	Downregulated	Bad while downregulated
miR-708 [91]	11	Downregulated	Bad while downregulated
miR-9-3 [97,98]	15	Downregulated	Good while upregulated
miR-143 [99,100]	5	Downregulated	Good while upregulated
miR-126 [63,106]	9	Downregulated	Bad while downregulated

Novel therapeutic directions

Since 2002, when Calin et al. discovered that miR-15 and miR-16 are downregulated in most CLL cases, there have been numerous studies on the implications of miRNAs in both malignant and non-malignant pathologies [17,109,110]. However, no miRNA has been included as a prognostic or predictive marker or as a therapeutic target in a guideline, thus miRNAs remain a niche. To our knowledge, the number of clinical trials involving CLL, and miRNAs is limited, and this could be secondary to the fact that CLL usually has an indolent evolution and already has a wide range of therapeutic options. But there are cases in which the disease exceeds therapeutic means and the answer to the heterogeneity of CLL manifestations may lie in the modulation of miRNAs.

Jurado-Camino et al. evaluated the innate immunity of 70 untreated patients diagnosed with CLL. Monocytes were found to be in an endotoxin-tolerant (ET) state, unable to perform their immune functions. The patients' monocytes were treated with lipopolysaccharides (LPS) for three hours, which caused a decrease in cytokine production. The basal cytokine levels were also determined and did not differ from healthy controls [111].

LPS stimulates NF-kB via TLR4, leading to the transcription of TNFα, IL-1β, IL-6, and IL-12 genes, and subsequently activates the anti-inflammatory response. ET of monocytes in humans and mice is associated with decreased expression of TARF-6 and IRAK-1 activity, through dysregulation of the TLR4-MYD88-IRAK axis [111].

Ten patients were randomly selected and assessed for miR-146 levels, which were overexpressed in monocytes, but not in lymphocytes. It was found that miR-146 inhibits IRAK1 and TRAF6 genes, inducing endotoxin tolerance, with the inability of monocytes to exert immune function, without any correlation with immunoparalysis or dysimmune status of CLL patients [111].

This study provides new insights into infection control in patients with CLL, the leading cause of death in CLL [112]. Assessment and modulation of miR-146 could decrease infection-related mortality, independent of immunoparalytic or dysimmune status.

Starting from the fact that CLL cells express interleukin-23 receptor (IL-23R), which is stimulated by the expression of IL-12Rβ1, to produce IL-23, which will lead to tumor proliferation, Matis et al. investigated 224 patients with CLL with Binet stage A and no therapeutic need. In the included cohort of patients miR-146b-5p downregulation was correlated with decreased time to need for treatment. Further, the expression of this miRNA was negatively correlated with IL-12Rβ1 expression, which led to increased production of IL-23 and tumor development. In vitro and in vivo administration of miR-146b-5p mimics decreased the number of Ki67+ cells and IL-12Rβ1-expressing cells. However, this was not validated for miR-146a-5p. MiR-146b-5p mimic reduced both tissue-infiltrating and circulating leukemic cells in NSG mice engrafted with CLL cells, through downregulation of IL-23R expression, but, as mentioned above, as well through downregulation of TRAF-6 and IRAK-1 [113]. MiR-146b-5p is a valuable therapeutic target and provides important information on the dysregulation of immune mechanisms that favor the development of CLL.

Chemoimmunotherapy with rituximab-fludarabine-cyclophosphamide was the standard of care for treatment-naïve, fit CLL patients [114]. The paradigm has changed, and the panel favors therapies that are better tolerated and lead to improved overall survival [2,115,116]. MiRNAs have a say in disease refractoriness to fludarabine. Manuela Ferracin and her collaborators evaluated the expression of 723 miRNAs in 29 CLL patients who received fludarabine single-agent therapy. They measured miRNAs’ expression before and five days after fludarabine administration and identified miR-148a, miR-222, and miR-21 highly expressed in non-responder patients, compared to the ones that obtained complete response. In vitro experiments on p53-mutant cell lines suggested that miR-21 and miR-222 inhibition may increase fludarabine sensitivity. CLL patients' ability to respond to fludarabine may be predicted based on the heterogenicity of miRNAs’ expression [117]. In another study by Zenz et al., miR-34a was downregulated in fludarabine-resistant CLL cells in a p53 defective pathway manner [118]. However, in Ferracin’s study, no difference in miR-34a expression was found between non-responders and responders, but it was noted that in three patients known to have del17p, miR-34 expression could not be detected [117].

The phase 3 clinical trial Rituximab in the Study of Relapsed Chronic Lymphocytic Leukemia (REACH) included 457 patients, out of which 37 had del17p and 52 had TP53 mutations. Further, those patients were divided into five categories, depending on the TP53 disruption level: wild-type TP53 and no del17p, single heterozygous TP53mut excluding TP53DN, only a detectable hemizygous del17p, hemizygous TP53mut and a hemizygous del17p (TP53mut/del17p), TP53DN or 1TP53mut. They evaluated the correlation between these groups of patients and the expression of miR-34. They found that not just the presence of TP53 mutations causes the downregulation of miR-34, but more importantly, the length of this abnormality. They also got conflicting results in the fourth group of patients, where despite the presence of TP53mut/del17p, they observed a high miR-34a expression and ones with heterozygous TP53mut with a low miR-34a expression, as well as in the first group of patients with no TP53 mutation or del17p and under-expressed miR-43a. These findings suggest that apart from TP53 disruption, other factors will influence miR-34 expression, like del1p36 [119]. Consequently, along with screening for TP53 mutation and del17p, miR-34a expression could assist in tailoring therapeutic approaches for CLL patients in the future.

In a prospective randomized trial that included 140 CLL patients without del17p treated with R-FC or dense-R-FC, the French Innovative Leukemia Organization, correlated the degree of rituximab-induced lymphodepletion with the expression of miR-125 and miR-532-3p [120]. In this case, miR-125b and miR-532-3p expression levels were inversely correlated with lymphodepletion rate. Despite these findings, no correlation was observed between the expression of these miRNAs and the clinical response assessed three months following R-FC treatment. A negative correlation was also found between CD20 expression on the surface of CLL cells and miR-125 and miR-532-3p, suggesting that these miRNAs, could influence the relationship between the expression level of CD20 and the depth of rituximab-induced lymphodepletion. This leads to the conclusion that miR-125b and miR-532-3p could serve as potential markers in CLL patients treated with rituximab, predicting the effectiveness of rituximab-induced lymphodepletion [120].

## Conclusions

Even though miRNAs regulate key processes involved in tumor development, such as apoptosis and proliferation, they are not used in clinical practice. Current studies indicate that identifying which miRNAs are relevant to CLL pathogenesis could provide potential biomarkers for diagnosis, prognosis, and therapeutic targets, leading to a better understanding of CLL biology and improving patients’ management and outcomes.
